# NBS1 is required for SPO11-linked DNA double-strand break repair in male meiosis

**DOI:** 10.1038/s41418-020-0493-4

**Published:** 2020-01-21

**Authors:** Bin Zhang, Zhenghui Tang, Lejun Li, Lin-Yu Lu

**Affiliations:** 1 Key Laboratory of Reproductive Genetics (Ministry of Education) and Women’s Reproductive Health Laboratory of Zhejiang Province, Women’s Hospital, Zhejiang University School of Medicine, Hangzhou, China; 2 Institute of Translational Medicine, Zhejiang University School of Medicine, Hangzhou, China

**Keywords:** Cell biology, Molecular biology

## Abstract

DNA double-strand breaks (DSBs) pose a serious threat to genomic stability. Paradoxically, hundreds of programed DSBs are generated by SPO11 in meiotic prophase, which are exclusively repaired by homologous recombination (HR) to promote obligate crossover between homologous chromosomes. In somatic cells, MRE11-RAD50-NBS1 (MRN) complex-dependent DNA end resection is a prerequisite for HR repair, especially for DSBs that are covalently linked with proteins or chemicals. Interestingly, all meiotic DSBs are linked with SPO11 after being generated. Although MRN complex’s function in meiotic DSB repair has been established in lower organisms, the role of MRN complex in mammalian meiotic DSB repair is not clear. Here, we show that MRN complex is essential for repairing meiotic SPO11-linked DSBs in male mice. In male germ cells, conditional inactivation of NBS1, a key component of MRN complex, causes dramatic reduction of DNA end resection and defective HR repair in meiotic prophase. NBS1 loss severely disrupts chromosome synapsis, generates abnormal chromosome structures, and eventually leads to meiotic arrest and male infertility in mice. Unlike in somatic cells, the recruitment of NBS1 to SPO11-linked DSB sites is MDC1-independent but requires other phosphorylated proteins. Collectively, our study not only reveals the significance of MRN complex in repairing meiotic DSBs but also discovers a unique mechanism that recruits MRN complex to SPO11-linked DSB sites.

## Introduction

DNA double-strand breaks (DSBs) are the most deleterious DNA damage in cells. DSBs can be repaired by either non-homologous end joining (NHEJ) or homologous recombination (HR) [1, 2]. The determinant step for HR repair is DNA end resection, which generates single-stranded DNA for strand invasion. DNA end resection requires the orchestration of several nucleases. A two-step, bidirectional model was proposed for this process [3]. Together with CTIP/Sae2, the MRE11-RAD50-NBS1/XRS2 (MRN/MRX) complex first utilizes MRE11’s endonuclease activity to cleave the 5′ strand away from the DSB. In the next step, the MRN complex generates short single-stranded DNA through the 3′ to 5′ exonuclease activity of MRE11, while EXO1 and DNA2 promote extended resection through their 5′ to 3′ exonuclease activities to generate long single-stranded DNA [4].

It has been shown that EXO1 and DNA2 can resect clean DSBs directly from the ends without MRE11’s activity [5–8]. However, since EXO1 or DNA2 cannot directly process DSBs that are covalently linked to proteins or chemicals, MRN complex-dependent endocleavage is particularly required for resection of this type of DSBs. In somatic cells, Topoisomerase II (TOP2) resolves DNA catenanes during DNA replication and transcription by transiently cutting and ligating DNA [9]. However, spontaneous abortive ligation causes TOP2 to be covalently linked to 5′ DNA ends, forming stable TOP2-linked DSBs [10, 11]. Such structures can be further induced by topoisomerase poisons such as etoposide. TOP2-linked DSBs are predominantly repaired through NHEJ, but this process requires TOP2 to be proteolyzed by proteasomes and the linkage between remaining TOP2 peptide and DNA to be cleaved by TDP2, generating clean DNA ends for LIG4-depedent NHEJ pathways [12–15]. TOP2-linked DSBs can also be repaired by HR. This process requires MRN complex to initiate DNA resection and remove TOP2-linked DNA, allowing EXO1 and DNA2 to further resect the DNA [16, 17]. Therefore, TDP2-dependent NHEJ pathway and MRN complex-dependent HR pathway function in parallel to repair TOP2-linked DSBs. Accordingly, MRE11 inactivation is synergistic to TDP2 loss regarding their effects on cellular sensitivity to etoposide [16].

Protein-linked DSBs are also generated in physiological conditions. In meiotic prophase, hundreds of programed DSBs are generated throughout all chromosomes by SPO11, a TOP2-like transesterase that is linked to the 5′ end of DSBs [18, 19]. Unlike in somatic cells, TDP2 loss in mice does not affect meiotic progression or fertility, suggesting that NHEJ is not used for repairing meiotic SPO11-linked DSBs [14]. This is consistent with the idea that DSBs in meiotic prophase are designated to be repaired by HR to promote obligate crossover between homologous chromosomes [20, 21]. Studies in multiple organisms have demonstrated that MRN/MRX complex-dependent removal of SPO11-oligonucleotide from DSB sites is required for meiotic DSB repair. In yeast, it has been established that all three proteins in MRX complex are required for resection of SPO11-linked DSBs in meiotic prophase [22], in addition to their roles in the formation of meiotic DSBs [23]. In *C. elegans*, MRE11 and RAD50 are required for the formation and resection of meiotic DSBs, but NBS1 is only required for resection [24–26].

In mice, knockout of each component of MRN complex results in embryonic lethality, preventing the study of the function of MRN complex in meiotic DSB repair. Some mutant mice of MRN complex are viable, allowing the examination of meiotic phenotypes in these mice (summarized in Table S1). However, no severe defect in meiotic DSB repair has been found in these mice. In *Mre11*^*ATLD1/ATLD1*^ mutant mice, mild meiotic phenotypes are present in both sexes [27–29], but only female mice are sub-fertile and male mice are fertile. The GAR motif mutant mice *Mre11*^*RK/RK*^ are viable and fertile [30]. For RAD50, *Rad50*^*S/S*^ hypomorphic mutant male mice have severe reduction of cellularity in testis, but meiotic progression is not blocked and the mice are fertile. Similar phenotype is observed in *Rad50*^*+/46*^ gain-of-function mutant male mice that are infertile, but meiotic progression is not blocked either [31]. For NBS1, *Nbs1*^*ΔB/ΔB*^ and *Nbs1*^*ΔC/ΔC*^ mice are fertile [32, 33]. For other *Nbs1* mutant mice, infertility is found only in females but not in males. *Nbs1*^*m/m*^ female mice completely lack oocytes, but the reasons are not clear [34]. In *Nbs1*^*−/−*^ mice rescued by human BAC containing NBS1 mutant *hNbs1*^*657Δ5*^ and *hNbs1*^*H45A*^, female mice are also devoid of oocytes. Meiosis in *hNbs1*^*657Δ5*^ female mice is arrested at pachytene stage with all chromosomes fully synapsed [35], which is different from impaired chromosome synapsis and zygotene stage meiotic arrest observed in mice with defective meiotic DSB repair, such as *Dmc1* KO [36, 37]. Conditional disruption of NBS1 during meiosis using *Spo11-Cre* does not lead to meiotic defects either [38]. Collectively, the roles of MRN complex in repairing meiotic SPO11-linked DSBs in mice are still poorly understood.

NBS1 was originally identified as the gene mutated in Nijmegen breakage syndrome, a rare disorder characterized by genomic instability, radiosensitivity, immunodeficiency, and increased cancer incidence [39, 40]. In cells from these patients, MRE11 and RAD50 fail to localize to the DSB sites [39]. Later studies reveal that NBS1 is also required for the nuclear localization of the MRN complex [39, 41]. A recent study has found that NBS1 senses CTIP phosphorylation and activates MRE11’s endonuclease activity [42]. Therefore, inactivation of NBS1 disrupts the function of the entire MRN complex in DNA repair. To examine the function of MRN complex in meiotic DSB repair in mice, we conditionally inactivate NBS1 in germ cells. NBS1 loss compromises the repair of SPO11-linked DSBs, disrupts chromosome synapsis, generates abnormal chromosome structures, and eventually leads to meiotic arrest and male infertility.

## Results

### NBS1 deficiency leads to etoposide sensitivity

**Fig. 1:**
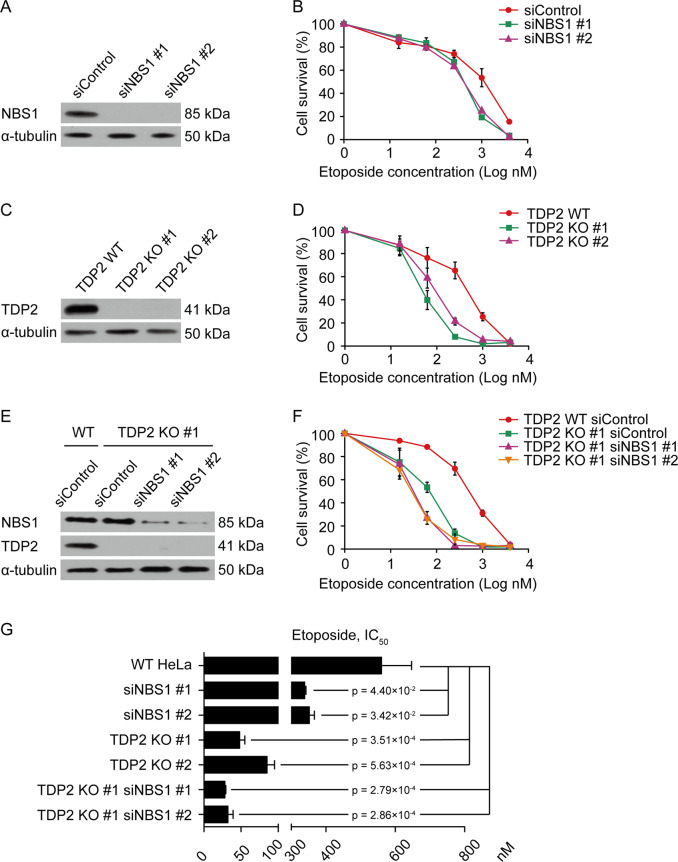
NBS1 deficiency leads to etoposide sensitivity. **a** Western blotting analyses of HeLa cells after NBS1 depletion by siRNA. α-tubulin was used as loading control. **b** Cell survival assays of NBS1-depleted HeLa cells with indicated doses of etoposide. **c** Western blotting analyses of TDP2 KO HeLa cells. α-tubulin was used as loading control. **d** Cell survival assays of TDP2 KO HeLa cells with indicated doses of etoposide. **e** Western blotting analyses of NBS1 protein level in TDP2 KO HeLa cells transfected with NBS1 siRNAs. α-tubulin was used as loading control. **f** Cell survival assays of TDP2 KO HeLa cells after NBS1 depletion with indicated doses of etoposide. **g** Summary of IC_50_ values (half maximal inhibitory concentration) of etoposide in the indicated HeLa cell lines. Error bars represent standard deviation, *n* = 3.

In order to examine the role of NBS1 in repairing TOP2-linked DSBs, we depleted NBS1 by siRNA in HeLa cells and tested their sensitivity to TOP2 poison etoposide (Fig. 1a). NBS1 depletion led to a significant reduction of cell viability after etoposide treatment (Fig. 1b, g). TOP2-linked DSBs can be processed by proteasome and TDP2 to promote NHEJ or processed by MRN to promote HR. In order to specifically examine NBS1’s role in HR repair of TOP2-linked DSBs, we inactivated NHEJ pathway by generating TDP2 KO in HeLa cells (Fig. 1c). Consistent with the idea that TOP2-linked DSBs are predominantly repaired through NHEJ, TDP2 KO cells are more sensitive to etoposide than NBS1 depletion (Fig. 1b, d, g). Interestingly, depletion of NBS1 further increased the sensitivity of TDP2 KO cells to etoposide, suggesting that NBS1 is indeed important for HR repair of TOP2-linked DSBs (Fig. 1e–g). In agreement with previous studies by MRE11 depletion [16, 17], ours results support the idea that TDP2-dependent and MRN-dependent pathways function in parallel to repair TOP2-linked DSBs. Our observations also suggest that NBS1 depletion is sufficient to disrupt the ability of MRN complex to repair TOP2-linked DSBs.

### NBS1 deficiency leads to male infertility

**Fig. 2:**
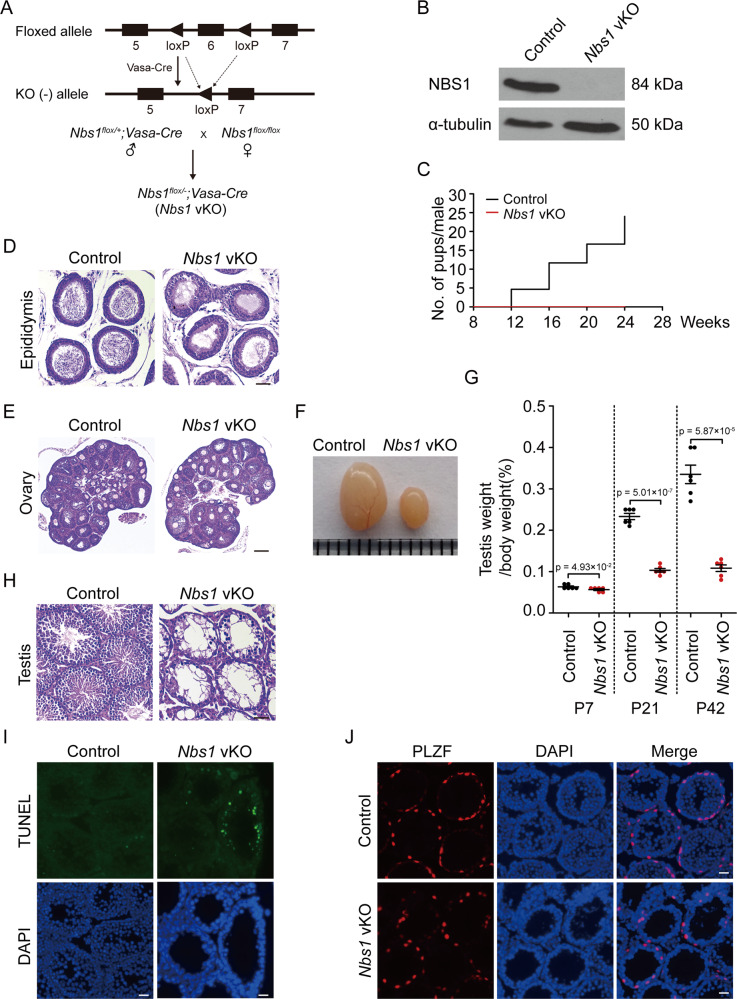
NBS1 deficiency leads to male infertility. **a** Schematic illustrations of mating strategies for obtaining *Nbs1* vKO mice. **b** Western blotting analyses of NBS1 in germ cells of *Nbs1* vKO male mice at postnatal day 21 (P21). α-tubulin was used as loading control. **c** Cumulative numbers of pups per male obtained by mating control or *Nbs1* vKO male mice with WT female mice. Three males of each genotype were used for mating. **d** H/E staining of paraffin sections of epididymis from control and *Nbs1* vKO male mice (P42). Scale bar, 200 µm. **e** H/E staining of paraffin sections of ovaries from control and *Nbs1* vKO female mice (P21). Scale bar, 200 µm. **f** Testes image of adult control and *Nbs1* vKO male mice. **g** Testis to body weight ratios of control and *Nbs1* vKO male mice (P7, P21, and P42). Error bars represent standard error of mean (SEM), *n* = 6. **h** H/E staining of paraffin sections of testes from control and *Nbs1* vKO male mice (P21). Scale bar, 200 µm. **i** TUNEL staining of testis frozen sections from control and *Nbs1* vKO mice (P21). The DNA was stained with DAPI. Scale bar, 20 µm. **j** Immunofluorescence staining analyses of PLZF in testis frozen sections from control and *Nbs1* vKO mice (P21). Scale bar, 40 µm. The DNA was stained with DAPI.

In meiotic prophase, programed SPO11-linked DSBs are generated, which are similar to TOP2-linked DSBs. To examine if MRN complex is required for repairing SPO11-linked DSBs during meiosis, we generated *Nbs1* germ cell conditional knockout mice using *Vasa-Cre* that starts to express in germ cells at embryonic day 15.5 (referred to as *Nbs1* vKO hereinafter) (Fig. 2a) [43]. In female mice, the Cre protein are expressed after SPO11-linked DSBs are generated, causing *Nbs1* vKO female mice not useful for the purpose of this study. However, in male mice, the Cre protein is expressed before meiotic prophase. Therefore, *Nbs1* vKO male mice are suitable tools to study the role of MRN complex in repairing SPO11-linked DSBs in meiosis.

Western blotting confirmed that NBS1 protein was successfully depleted in germ cells from *Nbs1* vKO testes (Fig. 2b). Fertility examination revealed that *Nbs1* vKO male mice were infertile (Fig. 2c). Consistently, no mature spermatozoa were found in epididymis of *Nbs1* vKO male mice (Fig. 2d). On the contrary, *Nbs1* vKO female mice were fertile and the number of oocytes was normal in ovaries (Fig. 2e). Interestingly, testis size and weight were dramatically reduced in *Nbs1* vKO male mice (Fig. 2f, g). In adult mice, no haploid cells were found in testis sections and apoptotic cells were significantly increased in *Nbs1* vKO male mice (Fig. 2h, i), suggesting that meiotic progression was arrested. However, no apparent defect was observed in spermatogonia that were positive for PLZF (promyelocytic leukemia zinc finger) (Fig. 2j). Therefore, NBS1 depletion in germ cells led to a specific defect in meiosis.

### NBS1 is required for chromosome synapsis and meiotic progression

**Fig. 3:**
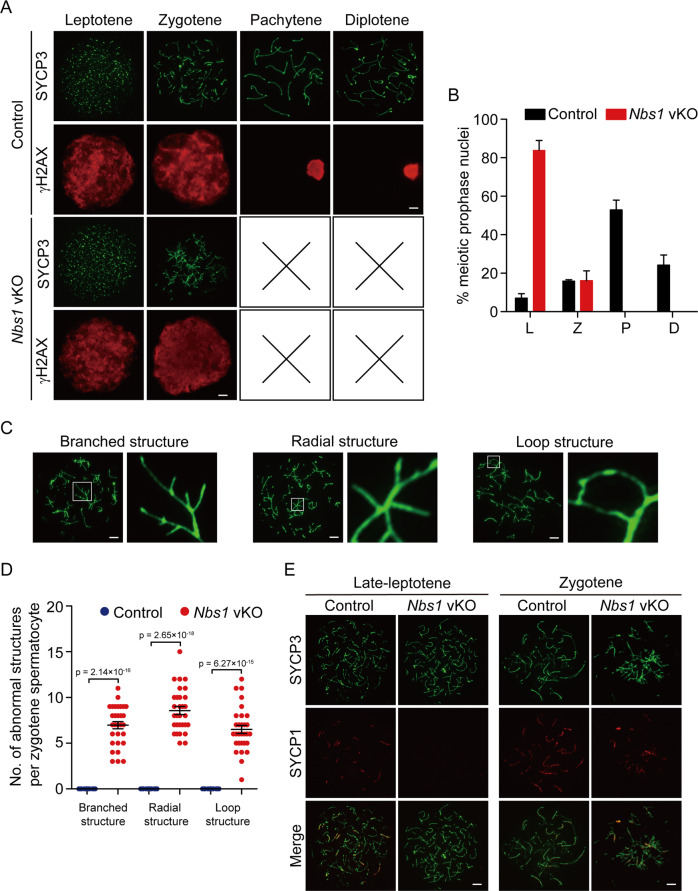
NBS1 is required for chromosome synapsis and meiotic progression. **a** Immunofluorescence staining of SYCP3 and γH2AX was used for analyses of stages of meiotic prophase in spermatocytes from control and *Nbs1* vKO mice. Scale bar, 5 µm. **b** Percentage of spermatocytes at each stage of meiotic prophase from control and *Nbs1* vKO mice. Error bars represent standard deviation, *n* = 3. L leptotene, Z zygotene, P pachytene, D diplotene. **c** Typical abnormal chromosome structures in *Nbs1* vKO zygotene spermatocytes. White boxes indicate the sites of abnormal chromosomes, and are magnified on the right. Scale bar, 5 µm. **d** Number of abnormal chromosome structures per zygotene spermatocyte from control and *Nbs1* vKO mice. Error bars represent SEM, *n* = 30. **e** Immunofluorescence staining of SYCP1 and SYCP3 were used for analyses of chromosome synapsis at late leptotene and zygotene stages of meiotic prophase from control and *Nbs1* vKO mice. Scale bar, 5 µm. Defective chromosome synapsis was observed in all *Nbs1* vKO spermatocytes (100%) but none of the control spermatocytes (0%).

To examine how meiotic progression was arrested in *Nbs1* vKO male mice, we examined the stages of meiotic prophase by SYCP3 and γH2AX immunostaining of spermatocytes. In control mice, all stages of meiotic prophase were identified. On the contrary, meiotic progression was arrested at zygotene stage in *Nbs1* vKO male mice (Fig. 3a, b). At zygotene stage, γH2AX signals were strong and covered the entire chromosomes, suggesting that SPO11-linked DSBs were produced normally. However, abnormal chromosome structures were observed. Most chromosomes were tangled and connected to each other randomly (Fig. 3c, d). In many occasions, several chromosomes were connected together at a small region and formed branched or radial structures. The chromosomes were sometimes connected to themselves and formed loop structures as well. This observation suggested that homologous chromosomes were not properly synapsed. Indeed, analysis of central components of synaptonemal complex SYCP1 revealed that chromosome synapsis was absent at late leptotene and was dramatically reduced at zygotene in *Nbs1* vKO male mice (Fig. 3e). Chromosome synapsis defects were observed in all *Nbs1* vKO spermatocytes but none of the control spermatocytes. Collectively, NBS1 loss in germ cells led to defective chromosome synapsis and abnormal chromosome structures at meiotic prophase.

### NBS1 is required for DNA end resection and HR repair at meiotic prophase

**Fig. 4:**
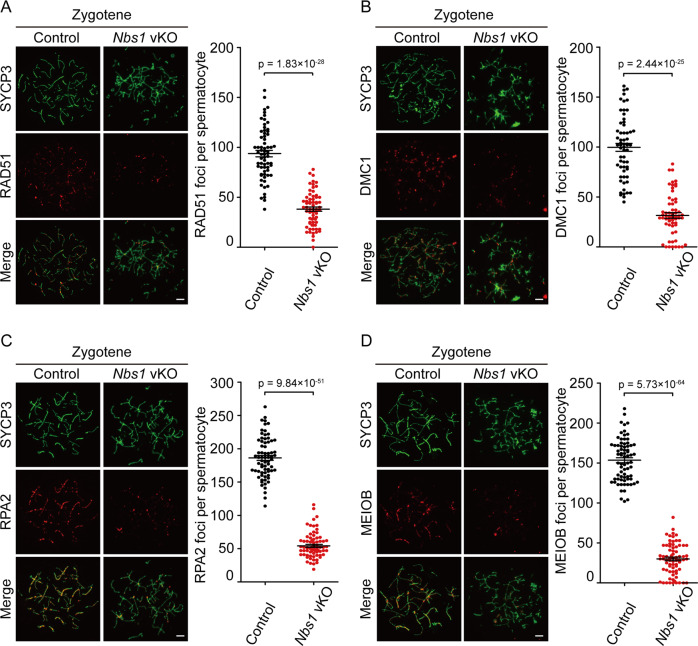
NBS1 is required for DNA end resection and HR repair at meiotic prophase. Surface spreads of control and *Nbs1* vKO mice were incubated with antibodies against RAD51 (**a**), DMC1 (**b**), RPA2 (**c**), and MEIOB (**d**). Representative surface spreads were shown on the left and the statistical charts were shown on the right. Scale bar, 5 µm. Error bars represent SEM, *n* ≥ 60.

Since no studies have suggested that NBS1 directly participates in chromosome synapsis, the abnormal chromosome structures in *Nbs1* vKO spermatocytes were likely a consequence of HR repair defect. Therefore, we investigated the HR repair status in these cells by examining the recruitment of RAD51 and DMC1, two key enzymes for HR repair in meiotic prophase. In zygotene spermatocytes from control mice, RAD51 and DMC1 were recruited and formed foci at chromosome axis, where DSBs were present. On the contrary, in zygotene spermatocytes from *Nbs1* vKO mice, RAD51 and DMC1 foci were dramatically reduced (Fig. 4a, b). The remaining foci were often observed at the region where abnormal chromosome structures were present, suggesting that they might contribute to the formation of abnormal chromosome structures in *Nbs1* vKO spermatocytes. Since DNA end resection is required for RAD51 and DMC1 loading, we further examined the status of single-stranded DNA-binding protein RPA2 and MEIOB (meiosis specific with OB-fold), which can reflect the amount of the single-stranded DNA produced by DNA end resection. In zygotene spermatocytes from control mice, RPA2 and MEIOB foci were present at the axis of each chromosome. However, RPA2 and MEIOB foci were significantly reduced in zygotene spermatocytes from *Nbs1* vKO mice (Fig. 4c, d). These observations suggested that NBS1 was important for resection of SPO11-linked DSBs at meiotic prophase.

### MDC1 is dispensable for NBS1’s function at meiotic prophase

**Fig. 5:**
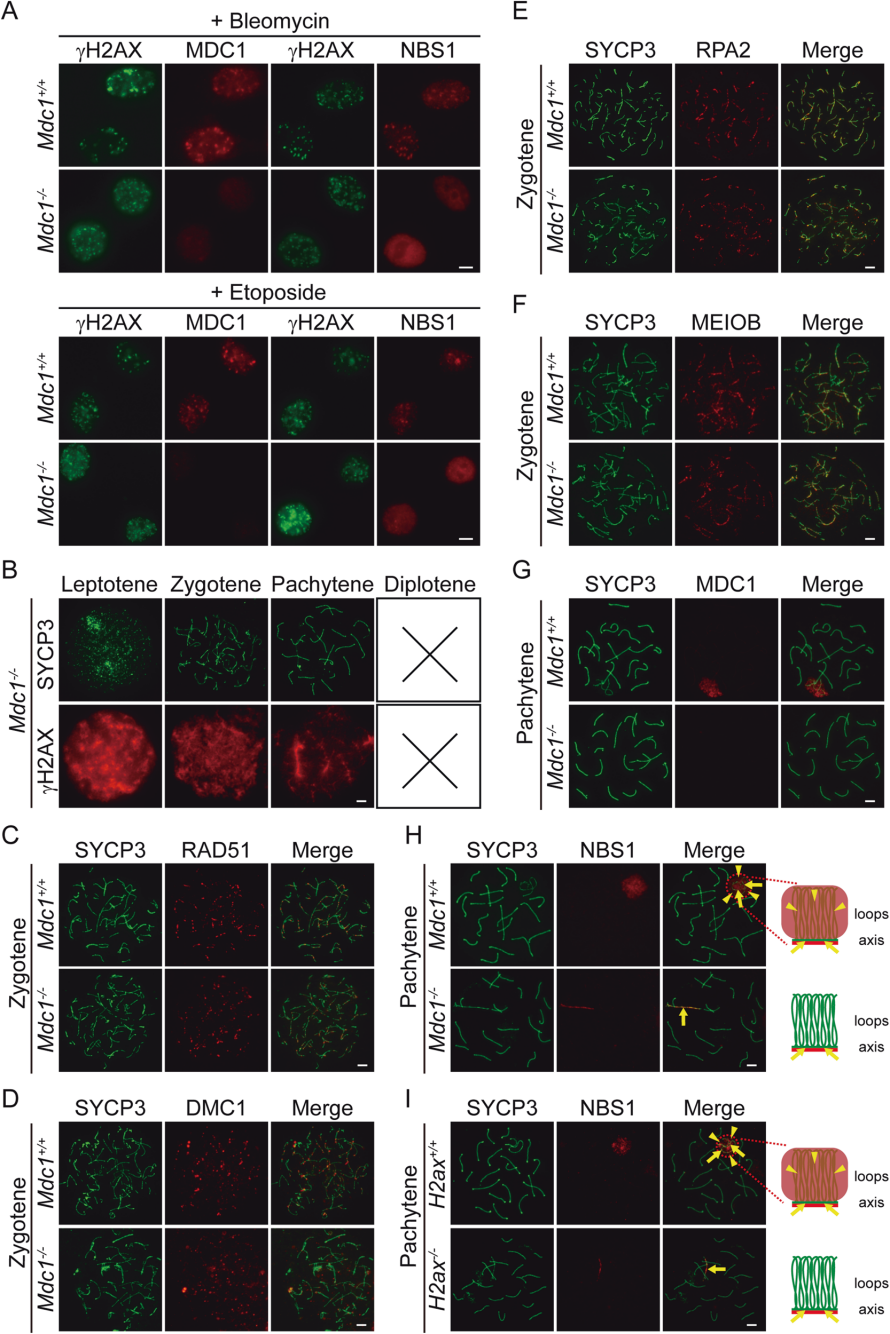
MDC1 is dispensable for NBS1’s function at meiotic prophase. **a** Immunofluorescence staining analyses of MDC1 and NBS1 in *Mdc1*^+/+^ and *Mdc1*^*−*/*−*^ MEFs after treatments of 10 µM bleomycin for 6 h or 5 µM etoposide for 2 h. γH2AX marks DNA damage sites. Scale bar, 10 µm. **b** Immunofluorescence staining of SYCP3 and γH2AX were used for analyses of stages of meiotic prophase in spermatocytes from *Mdc1*^*−*/*−*^ mice. Scale bar, 5 µm. Surface spreads of zygotene spermatocytes from *Mdc1*^+/+^ and *Mdc1*^*−*/*−*^ mice were incubated with antibodies against RAD51 (**c**), DMC1 (**d**), RPA2 (**e**), and MEIOB (**f**). Scale bar, 5 µm. **g** Localization of MDC1 in surface spreads of pachytene spermatocytes from *Mdc1*^+/+^ and *Mdc1*^*−*/*−*^ mice. Scale bar, 5 µm. **h** Localization of endogenous NBS1 in surface spreads of pachytene spermatocytes from *Mdc1*^+/+^ and *Mdc1*^*−*/*−*^ mice. Scale bar, 5 µm. Cartoons depicting proteins (red) localized on chromosome loops and axes (green) are shown on the right. Arrows mark chromosome axes and arrowheads mark chromosome loops. **i** Localization of endogenous NBS1 in surface spreads of pachytene spermatocytes from *H2ax*^+/+^ and *H2ax*^*−*/*−*^ mice. Scale bar, 5 µm. Cartoons depicting proteins (red) localized on chromosome loops and axes (green) are shown on the right. Arrows mark chromosome axes and arrowheads mark chromosome loops.

In somatic cells, the recruitment of NBS1 to DSB sites requires MDC1 (mediator of DNA damage checkpoint protein 1). NBS1 binds phosphorylated MDC1 and relocates to DSB sites along with MRE11 and RAD50 [44–49]. Indeed, NBS1 failed to form foci at bleomycin-induced DSB sites in *Mdc1* KO mouse embryonic fibroblasts (MEFs) (Fig. 5a). In addition, NBS1 foci were absent at etoposide-induced DSB sites in *Mdc1* KO MEFs (Fig. 5a), suggesting that NBS1 is also downstream of MDC1 for repairing TOP2-linked DSBs. Based on this observation, it is reasonable to speculate that NBS1 is downstream of MDC1 for repairing SPO11-linked DSBs at meiotic prophase. However, meiotic progression was arrested not at zygotene but at mid-pachytene in *Mdc1* KO spermatocytes (Fig. 5b). Therefore, meiotic progression was arrested at a much earlier stage in *Nbs1* vKO spermatocytes than in *Mdc1* KO spermatocytes. In addition, unlike in *Nbs1* vKO spermatocytes, the recruitment of RAD51, DMC1, RPA2, or MEIOB was normal in zygotene spermatocytes from *Mdc1* KO mice (Fig. 5c–f). These observations strongly suggested that NBS1 might not be downstream of MDC1 in repairing SPO11-linked DSBs.

To directly test if MDC1 regulates NBS1’s recruitment of SPO11-linked DSBs, we examined the localization of NBS1 in control and *Mdc1* KO spermatocytes. In pachytene spermatocytes, male sex chromosomes form a special structure known as the XY body. Due to the absence of homologous chromosomes, DSB repair is delayed on male sex chromosomes and DNA damage response proteins accumulate in the XY body [50]. In meiotic prophase, sister chromatids are organized as chromosome loops along chromosome axis where axial component of the synaptonemal complex SYCP3 is present [19, 51–53]. Some DNA damage response proteins, such as MDC1, are not restricted to chromosome axis where SPO11-linked DSBs are located [54], but spread to chromosome loops and cover the entire XY body (Fig. 5g). Although NBS1 could not be detected in zygotene spermatocytes, it was present in the XY body in pachytene spermatocytes (Fig. 5h). Similar to MDC1, NBS1 signal spread from chromosome axis to chromosome loops and cover the entire XY body (Fig. 5g–h). Interestingly, NBS1 signal was absent from chromatin loops but was retained at chromosome axis in the XY body in *Mdc1* KO spermatocytes (Fig. 5g–h). To double confirm this finding, we examined NBS1 localization in spermatocytes deficient for H2AX, the upstream binding partner of MDC1 at DSB sites. Similarly, NBS1 signal was absent from chromatin loops but was retained at chromosome axis in the XY body in *H2ax* KO spermatocytes (Fig. 5i). Therefore, MDC1 is required for spreading NBS1 to chromosome loops, but is dispensable for NBS1’s recruitment to the chromosome axis in the XY body. Since SPO11-linked DSBs locate at chromosome axis [54], it is likely that NBS1 is not downstream of MDC1 in repairing SPO11-linked DSBs.

### NBS1 is recruited to SPO11-linked DSBs by a phosphorylated protein

**Fig. 6:**
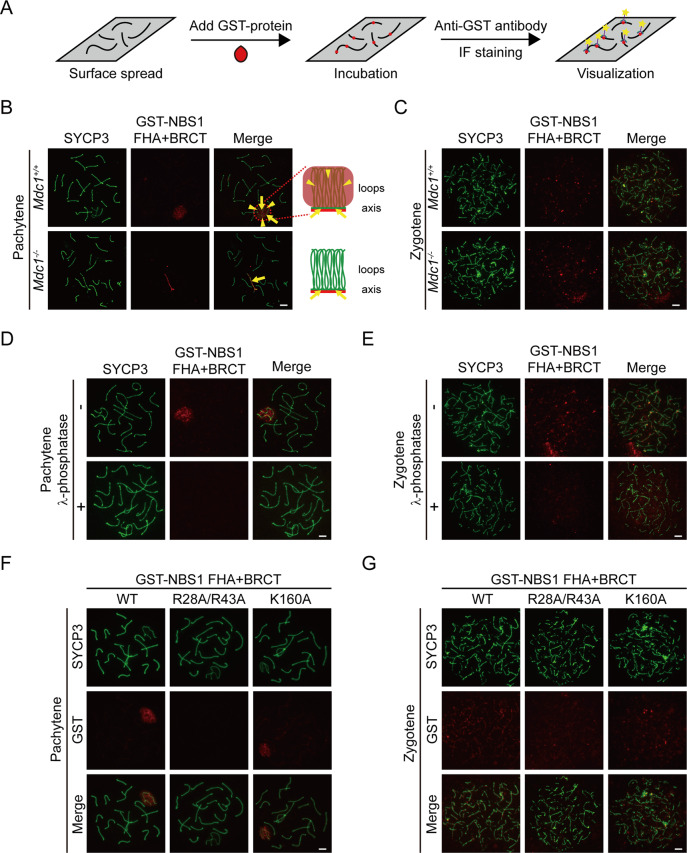
NBS1 is recruited to SPO11-linked DSBs by a phosphorylated protein. **a** Schematic representation of in vitro binding of recombinant proteins to surface spread of spermatocytes and visualization. **b** Localization of GST-tagged NBS1 FHA + BRCT domain protein at pachytene stage in surface spreads of spermatocytes from *Mdc1*^+/+^ and *Mdc1*^*−*/*−*^ mice. Scale bar, 5 µm. Cartoons depicting proteins (red) localized on chromosome loops and axes (green) are shown on the right. Arrows mark chromosome axes and arrowheads mark chromosome loops. **c** Localization of GST-tagged NBS1 FHA + BRCT domain protein at zygotene stage in surface spreads of spermatocytes from *Mdc1*^+/+^ and *Mdc1*^*−*/*−*^ mice. Scale bar, 5 µm. Localization of GST-tagged NBS1 FHA + BRCT domain protein at pachytene (**d**) and zygotene (**e**) stages in surface spreads of spermatocytes from wild-type mice with or without λ-phosphatase treatment. Scale bar, 5 µm. Localizations of wild type, FHA domain mutant (R28A/R43A), and BRCT domain mutant (K160A) forms of GST-tagged NBS1 FHA + BRCT domain proteins at pachytene (**f**) and zygotene (**g**) stages in surface spreads of spermatocytes from wild-type mice. Scale bar, 5 µm.

In somatic cells, the recruitment of NBS1 to DSBs through phosphorylated MDC1 binding requires its N-terminal folkhead-associated (FHA) and tandem BRCA1 C-terminal (BRCT) domains [44–49]. To examine how NBS1 is recruited to SPO11-linked DSBs, we utilized an in vitro binding system that we previously established to examine the localization of recombinant NBS1 protein fragments (Fig. 6a) [50]. Recombinant proteins of N-terminal FHA + BRCT domains of NBS1 were used since these domains fold together and are sufficient for phosphorylated MDC1 binding [48, 49]. Surface spread of spermatocytes was incubated with GST-tagged NBS1 FHA + BRCT domain proteins and the localization of the proteins was determined by immunofluorescent staining using an anti-GST antibody. Similar to endogenous NBS1, GST-tagged NBS1 FHA + BRCT domain proteins localized to the XY body in pachytene spermatocytes (Fig. 6b). Importantly, these proteins were present only at chromosome axis but not at chromosome loops in the XY body in *Mdc1* KO spermatocytes (Fig. 6b). Therefore, this method using GST-tagged NBS1 FHA + BRCT domain proteins fully recapitulated the staining pattern of endogenous NBS1. To our surprise, GST-tagged NBS1 FHA + BRCT domain proteins also localized at chromosome axis in zygotene spermatocytes (Fig. 6c), suggesting that this method is more sensitive than the anti-NBS1 antibody that we have used. The chromosome axis localization of these proteins was preserved in zygotene spermatocytes from *Mdc1* KO mice, further confirming the above observation in the XY body that MDC1 does not regulate the NBS1’s recruitment to SPO11-linked DSBs (Fig. 6c).

Interestingly, when surface spreads of spermatocytes were pretreated with λ-phosphatase to remove protein phosphorylation, the localization of GST-tagged NBS1 FHA + BRCT domain proteins was abolished in the XY body in pachytene spermatocytes and at chromosome axis in zygotene spermatocytes (Fig. 6d–e). Therefore, NBS1 is still recruited by phosphorylated proteins to SPO11-linked DSBs. Both FHA and BRCT domains can bind phosphorylated proteins [55, 56]. To examine which domain mediates the recruitment of NBS1, we generated GST-tagged NBS1 FHA + BRCT domain proteins with mutations at the phospho-binding pocket in each domain. The localization of these proteins in the XY body in pachytene spermatocytes and at chromosome axis in zygotene spermatocytes was abolished when the FHA domain was mutated (R28A/R43A), but was unaffected when the BRCT domain was mutated (K160A) (Fig. 6f, g). Taken together, these observations suggest that NBS1 binds to an unknown phosphorylated protein using its FHA domain and is recruited to SPO11-linked DSBs. The identity of this protein remains to be discovered.

## Discussion

**Fig. 7:**
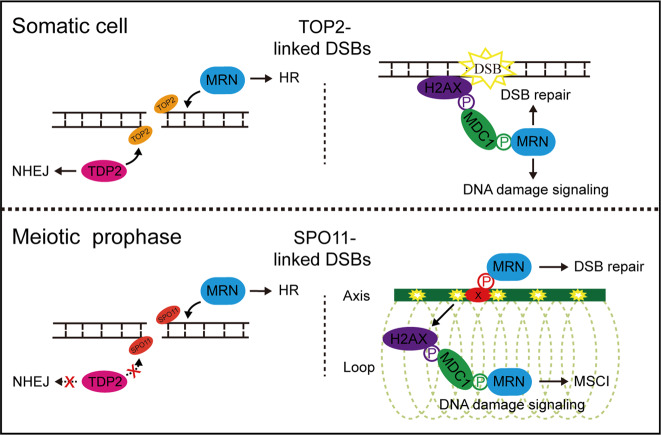
Working model: functions of MRN complex in protein-linked DSB repair in somatic cells and meiotic prophase. In somatic cells, TDP2-dependent NHEJ pathway and MRN complex-dependent HR pathway function in parallel to repair TOP2-linked DSBs. MRN complex is recruited to DSB sites by phosphorylated MDC1. In meiotic prophase, TDP2-dependent NHEJ pathway is inactivated by unknown mechanisms and MRN complex-dependent HR pathway is essential for repairing SPO11-linked DSBs. The recruitment of NBS1 to SPO11-linked DSB sites on meiotic chromosome axes is independent of MDC1 but requires another unknown phosphorylated protein. MDC1 is required for spreading NBS1 signals from DSB sites to chromosome loops in XY body to mediate meiotic sex chromosome silencing (MSCI).

In this study, we have shown that conditional inactivation of NBS1 in germ cells leads to meiotic arrest and male infertility. For the first time, our study has demonstrated that the MRN complex, like its homolog in lower organisms, is essential for repairing SPO11-linked DSBs in meiotic prophase in mammals (Fig. 7). This is consistent with the role of MRN complex in repairing TOP2-linked DSBs in mammalian somatic cells [16, 17]. We have shown that the MRN complex is required for DNA end resection and loading of key HR enzymes to SPO11-linked DSB sites in meiotic prophase. Importantly, we have found that the loss of MRN complex’s function leads to severe chromosome aberrations, which is one of the most severe meiotic phenotypes ever observed. It is noteworthy that DNA end resection and loading of key HR enzymes to SPO11-linked DSB sites are not completely abolished. As previously reported, NBS1 depletion does not affect the expression levels of MRE11 or RAD50, but compromises MRE11’s nuclear localization and recruitment of DSB sites [39, 41]. It is possible that NBS1 loss does not completely prevent the localization of MRE11 at DSB sites, which accounts for the residual resection and HR activity. Conditional inactivation of MRE11 in germ cells can be performed in future to examine this possibility. C1QBP, a recently identified binding partner of MRE11 and RAD50, might mediate the residual MRE11 activity on meiotic chromosomes in the absence of NBS1 [57].

Besides being important for repairing SPO11-linked DSBs, the NBS1 ortholog XRS2 in yeast is also required for the formation of meiotic SPO11-linked DSBs [23]. On the contrary, γH2AX signals at leptotene spermatocytes are indistinguishable between *Nbs1* vKO and control mice, suggesting that NBS1 is dispensable for the formation of meiotic SPO11-linked DSBs in mice. However, there is a slight chance that γH2AX signals in *Nbs1* vKO mice are not caused by SPO11-linked DSBs, which can tested by generating *Spo11*^*−/−*^*;Nbs1* vKO mice in future.

Our studies have suggested that the fertility phenotypes of viable MRN complex mouse models in previous studies cannot accurately reflect the MRN’s function in meiosis. Unlike our study, conditional disruption of NBS1 during meiosis using *Spo11-Cre* does not lead to meiotic defects [38]. Since the DSB repair occurs immediately after SPO11 expression and DSB generation, it is likely that NBS1 protein fails to be fully depleted by *Spo11-Cre* when DSB repair takes place. On the contrary, *Vasa-Cre* used in our study starts to express from embryonic day 15.5 and is expressed in all male diploid germ cells after birth, which is far ahead of the start of meiosis prophase at around day 7 after birth [43]. In addition, our study suggests that NBS1 is not required for the function of premeiotic germ cells, which ensures NBS1-deficient germ cells to enter meiosis and allows meiotic defects to manifest.

Although all viable MRN complex mutant mice in previous studies have normal fertility in males, female infertility was observed in some NBS1 mutant mice [34, 35]. Interestingly, examination one of them reveals pachytene arrest, prompting a possibility that NBS1 have a specific role at pachytene stage in females. In our mice, NBS1 protein is likely not yet depleted at pachytene stages in females. Therefore, our mice are not suitable for addressing this possibility. Future studies by conditional inactivation of NBS1 before meiotic prophase in female are needed. Nevertheless, normal female fertility in our mice suggests that NBS1 is not important for oocyte maturation and maintenance.

In this study, we have shown that chromosome synapsis in mice is severely compromised after NBS1 depletion, suggesting that MRN complex-dependent SPO11-linked DSB repair is required for chromosome synapsis. DSB repair and chromosome synapsis are two events that occur simultaneously in meiotic prophase, but their relationship and dependency are not conserved among different organisms. In yeast, DSB repair is required for chromosome synapsis [58, 59]. However, in *Drosophila* and *C. elegans*, DSB repair is dispensable for chromosome synapsis [60, 61]. In mice, like in yeast, chromosome pairing at telomere region precedes DSB formation [62]. However, by comparing the timing of γH2AX disappearance and chromosome synapsis, it is proposed that DSB repair precedes chromosome synapsis in mice [63]. Indeed, chromosome synapsis is disrupted in *Spo11* KO spermatocytes that lack DSB production or *Dmc1* KO spermatocytes that are defective in DSB repair [36, 37, 64, 65]. MRN complex-mediated DNA resection and removal of SPO11 are the first steps of meiotic DSB repair. Therefore, our study suggests that DSB resection precedes chromosome synapsis in mice, which strong supports the idea that DSB repair is important for chromosome synapsis in mice.

In somatic cells, the recruitment of NBS1 to DSB sites requires MDC1 [44–49]. In this study, we have shown that the recruitment of NBS1 to chromosome axis in meiotic prophase uses an MDC1-independent mechanism (Fig. 7). Besides MDC1, RAD17 is also phosphorylated and regulates early recruitment of NBS1 to DSB sites in somatic cells [66]. Although the localization in meiotic prophase is unclear due to unavailability of antibodies, RAD17 is a potential candidate that warrants further investigation. In addition, the unique mechanism regulating NBS1’s recruitment in meiotic prophase is reminiscent of our previous observation that the recruitment of BRCA1 to chromosome axis in the XY body also uses a mechanism distinct from that in somatic cells [50]. In meiotic prophase, SPO11-linked DSB sites are located at chromosome axis [54], where synaptonemal complex proteins as well as many other meiosis-specific proteins are present [67]. It is possible that the unique protein environment on chromosome axis leads a different mechanism for the recruitment of DNA repair proteins. In support of this idea, unlike on chromosome axis, the spreading of NBS1 to chromosome loops in the XY body still relies on MDC1, suggesting that the mechanism for recruiting DNA damage signaling proteins to chromosome loops remains similar to that in somatic cells. Previous studies have shown that MDC1 is required for spreading the DNA damage signaling from chromosome axis to chromosome loops to enforce meiotic sex chromosome silencing (MSCI) [68]. Our observation further indicates that NBS1 might also have a role in MSCI (Fig. 7). Future studies are needed to reveal if NBS1 is important for MSCI.

## Materials and methods

### Mice

All mice experiments were permitted by Zhejiang University Animal Care and Use Committee. *Nbs1*^*flox/flox*^ mice were gifts from Zhao-Qi Wang (Fritz Lipmann Institute, Germany). *Mdc1*^*−/−*^ and *H2ax*^*−/−*^ mice were gifts from Xiaochun Yu (City of Hope). *Nbs1*^*flox/flox*^ mice were crossed with *Vasa-Cre* transgenic mice (The Jackson Laboratory, 006954) to obtain *Nbs1*^*flox/+*^*;Vasa-Cre* male mice, which were then crossed with *Nbs1*^*flox/flox*^ female mice to obtain *Nbs1*^*flox/−*^*;Vasa-Cre* (*Nbs1* vKO) mice. Genotyping was performed by PCR with tail genomic DNA. Forward primer 1 (5′-AGGTAATCAATCACTGTGCCTTCTA-3′) and reverse primer 1 (5′-ACAATACAGTGACTCCTGGAGGCAA-3′) were used for detecting *Nbs1* wild-type and the floxed allele. Forward primer 2 (5′-CAGGATGATGTCCCCATTCAGTTAG-3′) and reverse primer 1 were used for detecting KO allele. *Vasa-Cre* allele was detected by Forward primer 3 (5′-GTCGCCTGATGCTATTTGTTGTCC-3′) and reverse primer 2 (5′-AGGATGATGACCAGGATGTAGTTGT-3′).

### Antibodies

The following antibodies were purchased: anti-hNBS1 (Proteintech, 55025-1-AP), anti-mNBS1 (Abcam, ab32074), anti-TDP2 (Proteintech, 12203-1-AP), anti-α-tubulin (Genscript, A01410-100), anti-PLZF (R&D, AF1356), anti-γH2AX (Millipore, 05-636), mouse-anti-SYCP3 (Santa cruz, sc-74569), rabbit-anti-SYCP3 (Proteintech, 23024-1-AP), anti-SYCP1 (Novus, NB300-229SS), anti-RPA2 (Abcam, ab2175), anti-RAD51 (Santa Cruz, sc-8349), anti-DMC1 (Proteintech, 13714-1-AP). Anti-MDC1 and anti-GST antibodies were gifts from Xiaochun Yu (City of Hope), and anti-MEIOB antibody was a gift from Mengcheng Luo (Wuhan University).

### Cell culture and transfection

HeLa cells and MEFs were cultured in DMEM medium (Thermo Fisher) supplemented with 10% fetal bovine serum (FBS) and 1% penicillin and streptomycin at 37 °C with 5% CO_2_. NBS1 siRNAs (GenePharma) were transfected with lipofectamine 3000 (Thermo Fisher) according to the manufacturer’s instructions. After 48 h of transfection, cells were treated with etoposide or collected for experiments. The NBS1 siRNAs sequences are as follows: #1: 5′-AGAAACGUGAACUCAAGGA-3′; #2: 5′-AGGAAGAUGUCAAUGUUAG-3′.

### Generation of TDP2 KO HeLa cell lines

TDP2 KO HeLa cells were generated by CRISPR/Cas9 technology. TDP2 guide RNAs were cloned into PX459 V2.0 plasmids (gifts from Feng Zhang, Addgene 62988) and they were transfected in HeLa cells. After that, cells were selected by 1 µg/ml puromycin for 2 days. Individual clones were picked by serial dilutions and then were verified by western blotting. The sequences of guide RNAs against TDP2 are as follows: sgRNA#1: 5′-TCTGTCAGAGAGGGCTCGAG-3′; sgRNA#2: 5′-TTTAGGTAGCTATAATATGG-3′.

### Cell survival assay

A total of 600 cells per well were seeded in 12-well plates and 24 h later they were treated with etoposide for 3 h. Culture medium was changed every day. After 7 days, cells were fixed by 4% PFA and stained with 0.5% crystal violet. Stained cells were dissolved in 10% acetic acid solution for 3 h and the relative growth was measured by the absorbance at 595 nm.

### Germ cell enrichment

Tunica albuginea was removed from testes and seminiferous tubules were digested in 2 mg/ml type IV collagenase for 15 min at 37 °C. Cell pellet was collected and digested with TrypLE Express (Thermo Fisher) for 10 min at 37 °C. The digestion was stopped by adding DMEM medium with 10% FBS and was then centrifuged at 400 g for 2 min. The cell pellet was resuspended and seeded in a 10-cm dish and cultured in DMEM medium (Thermo Fisher) supplemented with 10% FBS and 1% penicillin and streptomycin at 37 °C with 5% CO_2_. One hour later, most cells in the supernatant were germ cells and were collected for western blotting.

### Western blotting

Cell lysates were extracted by NETN300 (300 mM NaCl, 0.5 mM EDTA, 0.5% (v/v) NP-40, 20 mM Tris-HCl pH 8.0). Target proteins were separated by SDS-PAGE and were transferred onto PVDF membranes by Trans-Blot Turbo (Bio-Rad). PVDF membranes were blocked by 5% milk in TBST for 30 min, blotted with primary antibody overnight, and then blotted with secondary antibody for 1 h. The ECL substrates were then added for detection using chemiluminescence system.

### Surface spread of spermatocytes

Tunica albuginea was removed from testes and seminiferous tubules were digested by 1 mg/ml type IV collagenase for 30 min. Cells were collected and were incubated with buffer 1 (30 mM Tris-HCl pH 8.2, 50 mM sucrose, 17 mM sodium citrate) for 10 min at room temperature. Cell pellet was then collected at 400 g for 1 min and was resuspended in buffer 2 (100 mM sucrose) for 5 min at room temperature. The cell suspension was added with the same volume of buffer 3 (1% PFA, 0.15% Triton X-100, 10 mM sodium borate, pH 9.2) before being spread onto slides and air-dried overnight.

### Immunofluorescence staining

Frozen sections were treated with 0.5% Triton X-100 for 10 min and were incubated with primary antibodies overnight. Sections were washed with PBS three times and were then incubated with secondary antibodies for 3 h. MEFs were pretreated with 0.5% Triton X-100 for 10 s and then fixed by 4% PFA for 10 min. Surface spread of spermatocytes was treated with 0.5% Triton X-100 for 2 min. For MEFs and surface spreads of spermatocytes, primary and secondary antibodies were incubated for 1 h and 30 min, respectively. TUNEL staining was performed using in situ cell death detection kit (Roche).

### In vitro binding of recombinant proteins to surface spread of spermatocytes

C-terminal GST-tagged NBS1 FHA + BRCT domain (aa 1-330) recombinant proteins were expressed in sf9 cells using bac-to-bac baculovirus expression system according to the manufacturer’s instructions. Recombinant proteins were purified by glutathione sepharose and were dialyzed in PBS. For removing protein phosphorylation, surface spread of spermatocytes was treated with 1000 units of λ-phosphatase (NEB). Overall, 20 nmol recombinant protein was added to 5% goat serum in PBS and was incubated with surface spreads for 1 h. The slides were then washed with PBS and immunofluorescence staining was performed by using anti-GST antibody.

### Statistical analyses

Two-tailed unpaired student’s *t* test was used to evaluate statistical significance for all experiments.

## Supplementary information

Table S1
